# Heightened risk of fatal police violence in and around reservations for American Indian/Alaska Native peoples in the United States

**DOI:** 10.1073/pnas.2521002123

**Published:** 2026-03-09

**Authors:** Gabriel L. Schwartz, Theresa Rocha Beardall, Jaquelyn L. Jahn

**Affiliations:** ^a^Health Management and Policy Department and the Urban Health Collaborative, Drexel University Dornsife School of Public Health, Philadelphia, PA 19104; ^b^Sociology Department, University of Washington, Seattle, WA 98195; ^c^Epidemiology and Biostatistics Department and the Ubuntu Center on Racism, Global Movements, and Population Health Equity, Drexel University Dornsife School of Public Health, Philadelphia, PA 19104

**Keywords:** Indigenous health, police violence, Native American reservations, mortality

## Abstract

Fatal police violence is a public health problem in the United States, with large racial and spatial inequities that remain understudied. Indigenous people, for example, experience some of the highest rates of fatal police violence of any racial/ethnic group. Residents of American Indian/Alaska Native (AIAN) reservations may be at particular risk, given geographically specific police surveillance, jurisdictional mazes, and structural disinvestment. Yet little quantitative work has examined fatal police violence against Indigenous peoples in the United States, including by reservation geography. We examined whether AIAN people experience higher rates of fatal police violence in and around reservations at the population level. We analyzed data on all AIAN people killed by police (n = 203, 2013–2024) from the Mapping Police Violence database. We first summarized their geographic distribution and characteristics using descriptive statistics. We then estimated rate ratios using quasi-Poisson regression models with population offsets, comparing AIAN peoples’ fatal police violence rates in and around reservations versus farther away. We find that fatal police violence against AIAN people is strongly concentrated in and around reservations: 73% of AIAN deaths occurred on or within 10 miles of reservations, despite only 39 to 51% of the AIAN population living there. Those areas’ residents were 1.60 to 5.84 times more likely to be killed by police than those living farther away, depending on location and choice of population offsets. Sensitivity analyses indicated these elevated risks could not plausibly be explained away by differential racial/ethnic misclassification. A coordinated public health response to police violence is urgently needed in Indian Country.

## Significance

One in 1800 Indigenous men in the United States will die from fatal police violence if current rates hold. We find that this risk is overwhelmingly concentrated in and around reservations, where structural disinvestment and unique policing models appear to put Indigenous peoples in harm’s way. We also show that the types of officers responsible for fatal police violence in these areas (mostly federal, state, and tribal) differ dramatically from those of responsible officers elsewhere (mostly municipal and county), as do the reasons police give for stops in and around reservations. We thus quantitatively demonstrate the geographic and policy areas where police violence interventions are most needed to protect Indigenous communities.

Fatal police violence in the United States is a public health problem marked by profound racial and spatial inequities (1). Indigenous people, for example, experience some of the highest population-based rates of fatal police violence of any racial/ethnic group (2). However, few national studies have examined fatal police violence against Indigenous peoples systematically, including where rates of this violence are most severe and what that can reveal about its potential policy drivers (3). This study addresses that gap by focusing on how fatal police violence against Indigenous peoples varies across reservation geographies, elucidating how historical and structural features of US governance shape Indigenous peoples’ exposure to police violence across space.

This research is possible because community data initiatives have arisen to track police violence deaths as public health data, enabling a growing body of research on their distribution and consequences for mental and physical health (4–6). Scholars and community advocates have used these data to highlight extreme racial and ethnic inequities in rates of fatal police violence rates and further our understanding of the ways structural racism shapes police violence risk. Such studies have revealed, for example, that cities with seemingly low overall police violence death rates can mask the most extreme racial inequities (1); demonstrated that Black people can face a heightened risk of fatal police violence in segregated, predominantly White neighborhoods (7); and estimated that Indigenous men and boys in the United States experience rates of fatal police violence that are between 1.2 and 1.7 times higher than their White counterparts (2).

## Pathways and Structures.

A key concept in the social science literature that is instructive for thinking about why reservations might matter is conceptualizing them as products of “settler colonialism”—i.e., conceptualizing the United States as “settler-colonial” with respect to First Nations (8, 9). By “settler-colonialism,” scholars mean (at a high level) that, rather than attempting to rule over North American lands from a distant metropole, settlers moved onto Indigenous lands *en masse* while constructing a political order that secured non-Native occupation (i.e., the often violent transfer of lands from Indigenous peoples to settlers, including Native communities’ forcible removal) and eroded Indigenous authority over land, law, and resources (10). These governance arrangements then have enduring effects on Indigenous peoples’ health; economic, social, and physical mobility; and safety (11–14).

Settler-colonial governance structures are most visible in and around reservations. Two features of reservations in particular A) demonstrate their unique position and B) make them potentially high-risk spaces for Indigenous residents with respect to police violence.

First, law enforcement on reservations operates differently than on nonreservation lands (8, 13). Federal, state, county, municipal, and tribal police may all have overlapping jurisdiction to police a reservation (depending on the crime, who committed it, and in what state), creating dense but weakly coordinated systems. Partly as a result of this jurisdictional fragmentation, Indigenous scholars have documented persistent racial profiling, harassment, and surveillance by non-Indigenous police officers at reservation borders (right where local law enforcement officers’ jurisdiction often begins) (15–17). Reservation residents accordingly report feeling as though Indigenous communities, movement, and land use are treated as administrative problems to be contained by local police through aggressive and discretionary enforcement (15, 16). These dynamics mirror what Rocha Beardall calls “sovereignty threat,” a form of minority-threat logic that authorizes anti-Indigenous state violence (17). And areas just outside of reservations (“reservation borderlands”) may be affected, too, given that these are where racial profiling of those entering and leaving reservations is occurring, and because many Indigenous communities geographically straddle reservation borders, with frequent coming and going (18).

Second, federal and state policy, coupled with chronic underinvestment, may deepen Indigenous peoples’ risks of interactions with police. Decades of fragmented governance between tribes and different levels of the US government–as well as the abandonment of treaty obligations and underfunding of health and education programs on reservations–have entrenched poverty, underfunded schools, and restricted access to public goods in many AIAN communities (12, 14, 19, 20). These inequities drive contact with the criminal legal system through cycles of poverty, illness, and mental health crisis (21, 22). Chronic underinvestment in the Indian Health Service further magnifies the danger by constraining emergency medical response, increasing the likelihood that violent police encounters on or near reservations will be fatal (14, 23). Together, these conditions may produce an epicenter of risk where state power and medical underinvestment converge.

Reservations and the borderlands around them are thus a crucial area of focus where settler-colonial logics, as well as their attendant policies, may impose heightened risks of fatal police violence on the AIAN people who live there. To our knowledge, no national study has quantified these risks at the population level, limiting the development of robust policy responses.

## The Present Study.

We use data from the Mapping Police Violence database (24) and the US Census to examine whether AIAN people living on or near reservations face elevated rates of fatal police violence compared to those living farther away. To do so, we first map these deaths’ geographic distribution, then use quasi-Poisson regression models to calculate rate ratios at the Census block group level, with areas far from reservations serving as our reference group and with our outcome defined as the cumulative count of AIAN police violence deaths within each block group across the study period. Offsets in our Poisson models (effectively, our rate denominators) are counts of each block group’s AIAN population, with multiple definitions used (single-race only vs. multiracial) to reflect the plausible range of rates depending on how the AIAN population might be differently defined; see Discussion. Sensitivity analyses also controlled for population density or urbanicity.

Importantly for the below, we initially break the borderlands around reservations into two buffer zones: within 5 miles of reservations [capturing the zone immediately outside reservations, where racial profiling might be most extreme; as well as “checkerboarded” pockets of nonreservation lands surrounded by reservations (13, 25)], and between 5 and 10 miles of reservations (capturing nearby towns that may also experience racial profiling and heightened police presence, especially given these communities’ social, economic, and family reservation ties) (18). Those living >10 miles (“far”) from a reservation are our reference group. In sensitivity analyses, we explore alternative distances.

## Results

### Descriptives.

From 2013 to 2024, MPV recorded 203 AIAN people killed by police. These people were overwhelmingly male, with the majority killed in Western states. Decedents were on average 33 years old, though they ranged in age from 14 to 65 (Table 1).

**Table 1.** Characteristics of AIAN decedents killed by police

Variable	Overall (n = 203)	On a reservation (n = 82)	Within 5 mi (n = 35)	Within 10 mi (n = 31)	Not near a reservation (n = 55)
*Sex*					
Female	12 (5.9%)	4 (4.9%)	2 (5.7%)	1 (3.2%)	5 (9.1%)
Male	188 (92.6%)	76 (92.7%)	32 (91.4%)	30 (96.8%)	50 (90.9%)
Missing	3 (1.5%)	2 (2.4%)	1 (2.9%)	0 (0%)	0 (0%)
*Age*					
Mean (SD)	33.2 (10.3)	33.7 (10.5)	32.4 (8.54)	33.2 (13.2)	32.9 (9.51)
*Census Region*					
Midwest	42 (20.7%)	16 (19.5%)	4 (11.4%)	5 (16.1%)	17 (30.9%)
Northeast	3 (1.5%)	0 (0%)	0 (0%)	1 (3.2%)	2 (3.6%)
South	30 (14.8%)	23 (28.0%)	3 (8.6%)	2 (6.5%)	2 (3.6%)
West	128 (63.1%)	43 (52.4%)	28 (80.0%)	23 (74.2%)	34 (61.8%)
*Responsible Agency Type*					
Federal	16 (7.9%)	13 (15.9%)	0 (0%)	0 (0%)	3 (5.5%)
State	17 (8.4%)	9 (11.0%)	1 (2.9%)	3 (9.7%)	4 (7.3%)
Tribal	23 (11.3%)	22 (26.8%)	1 (2.9%)	0 (0%)	0 (0%)
County	38 (18.7%)	13 (15.9%)	6 (17.1%)	5 (16.1%)	14 (25.5%)
Local/Municipal	109 (53.7%)	25 (30.5%)	27 (77.1%)	23 (74.2%)	34 (61.8%)
*Year*
2013	6 (3.0%)	3 (3.7%)	2 (5.7%)	1 (3.2%)	0 (0%)
2014	10 (4.9%)	3 (3.7%)	0 (0%)	4 (12.9%)	3 (5.5%)
2015	14 (6.9%)	4 (4.9%)	1 (2.9%)	1 (3.2%)	8 (14.5%)
2016	24 (11.8%)	10 (12.2%)	6 (17.1%)	1 (3.2%)	7 (12.7%)
2017	28 (13.8%)	6 (7.3%)	6 (17.1%)	9 (29.0%)	7 (12.7%)
2018	22 (10.8%)	8 (9.8%)	3 (8.6%)	4 (12.9%)	7 (12.7%)
2019	13 (6.4%)	5 (6.1%)	4 (11.4%)	0 (0%)	4 (7.3%)
2020	15 (7.4%)	6 (7.3%)	3 (8.6%)	1 (3.2%)	5 (9.1%)
2021	16 (7.9%)	8 (9.8%)	2 (5.7%)	2 (6.5%)	4 (7.3%)
2022	22 (10.8%)	11 (13.4%)	2 (5.7%)	3 (9.7%)	6 (10.9%)
2023	18 (8.9%)	11 (13.4%)	2 (5.7%)	4 (12.9%)	1 (1.8%)
2024	15 (7.4%)	7 (8.5%)	4 (11.4%)	1 (3.2%)	3 (5.5%)
*Police-reported reason for encounter*					
Domestic Disturbance	19 (9.4%)	7 (8.5%)	5 (14.3%)	1 (3.2%)	6 (10.9%)
Mental Health/Welfare Check	18 (8.9%)	5 (6.1%)	5 (14.3%)	4 (12.9%)	4 (7.3%)
None/Unknown	30 (14.8%)	16 (19.5%)	2 (5.7%)	6 (19.4%)	6 (10.9%)
Other Crimes Against People	6 (3.0%)	1 (1.2%)	2 (5.7%)	1 (3.2%)	2 (3.6%)
Other Nonviolent Offense	30 (14.8%)	13 (15.9%)	5 (14.3%)	2 (6.5%)	10 (18.2%)
Part 1 Violent Crime	41 (20.2%)	16 (19.5%)	6 (17.1%)	8 (25.8%)	11 (20.0%)
Person with a Weapon	37 (18.2%)	16 (19.5%)	5 (14.3%)	6 (19.4%)	10 (18.2%)
Traffic Stop	22 (10.8%)	8 (9.8%)	5 (14.3%)	3 (9.7%)	6 (10.9%)
*Allegedly Armed (Whether reported to be armed in news or police reports)*					
Unarmed	29 (35.4%)	12 (34.3%)	10 (32.3%)	21 (38.2%)	72 (35.5%)
Armed	53 (64.6%)	23 (65.7%)	21 (67.7%)	34 (61.8%)	131 (64.5%)

The law enforcement agencies responsible for killing AIAN decedents varied by area. On reservations, federal and state agencies were responsible for 27% of killings, compared to 2.9 to 7.3% in other areas. Indeed, federal, state, and tribal law enforcement agencies were collectively responsible for the majority of on-reservation police violence deaths (54%). In contrast, local/municipal police were the overwhelming driver of police violence deaths in the borderlands around reservations, at 74 to 77% depending on spatial buffer distance, compared to 30.5% on reservations and 62% far from reservations (Table 1).

Geographic discrepancies were also present when examining the reasons police reported for initially interacting with decedents. On reservations and in some borderland areas, no reason was given for close to 20% of lethal police stops, compared to only 11% in areas far from reservations. The rate at which decedents were armed, however (as reported by police), was roughly equivalent across geography.

Finally, a much higher percentage of fatal police violence deaths occurred in and around reservations than we would expect given the AIAN population’s geographic distribution (Table 2). Between 49 and 61% of the AIAN population lived >10 miles from a reservation, depending on how that population was defined, but only 27% of AIAN fatal police violence deaths occurred in those areas. That is, 73% of AIAN people killed by police were on reservations or within 10 miles of them.

**Table 2.** Geographic distribution of AIAN police violence deaths and population

Place	Single-race AIAN population*		Multiracial AIAN population*		AIAN police violence deaths		Cumulative raw rates per 100,000	
	*N*	*%*	*N*	*%*	*N*	*%*	*Single Race*	*Multiracial*
On a reservation	853,115	29.14	1,025,994	19.78	82	40.39	9.6	8.0
Within 5 mi	374,088	12.78	565,729	10.91	35	17.24	9.4	6.2
Within 10 mi	253,588	8.66	445,269	8.58	31	15.27	12.2	7.0
Not near a reservation	1,447,294	49.43	3,150,119	60.73	55	27.09	3.8	1.7
*Total*	2,928,085	100	5,187,111	100	203	100		

*Note*: Death data for American Indian and Alaska Native decedents (2013–2024) are drawn from the Mapping Police Violence database. Populations within each geographic area were approximated by multiplying the population of each Census block group (per the 2010 Census) by the proportion of that block group covered by a reservation or specific area around a reservation, then summing across block groups. Reservations in Hawai’i are excluded, as Native Hawaiians are considered part of the “Native Hawaiian or Pacific Islander” category within the Mapping Police Violence database (and thus the policing of Native Hawaiian reservations are not expected to disproportionately affect non-Hawaiian American Indian or Alaskan Native peoples). “Single-Race” refers to the population of people who chose AIAN as their only racial identity on the US Census; “multiracial” includes both those who selected only AIAN and those who selected AIAN in combination with any other racial or ethnic group.

### Modeling.

In unadjusted models, AIAN people on reservations faced rates of fatal police violence that were between 1.95 (when using single-race denominators) and 3.55 (when using multiracial denominators) times higher than AIAN people far from reservations (>10 miles). Relative rates for the borderlands around reservations were also elevated, with rate ratios of 2.71 to 3.73. Results were robust to adjustment for population density or for rural–urban status, though rate ratios were modestly attenuated (Table 3).

**Table 3.** Estimated fatal police violence rate ratios from quasi-Poisson regression models

Denominator	Place	Unadjusted			Adjusted for Pop. Density			Adjusted for USDA Rurality		
		*RR*	*P*	*95% CI*	*RR*	*P*	*95% CI*	*RR*	*P*	*95% CI*
Single-race AIAN	Reservation	1.95	<0.001	(1.49, 2.56)	1.60	0.001	(1.20, 2.14)	1.62	0.004	(1.17, 2.26)
	Buffer: 5 mi	2.71	<0.001	(1.95, 3.73)	2.44	<0.001	(1.75, 3.37)	2.44	<0.001	(1.69, 3.48)
	Buffer: 10 mi	3.05	<0.001	(2.10, 4.35)	2.90	<0.001	(2.00, 4.15)	3.09	<0.001	(2.05, 4.56)
Multiracial AIAN (single or multiracial)	Reservation	3.55	<0.001	(2.77, 4.57)	2.93	<0.001	(2.25, 3.84)	2.71	<0.001	(2.01, 3.66)
	Buffer: 5 mi	3.87	<0.001	(2.86, 5.19)	3.55	<0.001	(2.61, 4.78)	3.36	<0.001	(2.41, 4.65)
	Buffer: 10 mi	3.73	<0.001	(2.65, 5.17)	3.60	<0.001	(2.55, 5.01)	3.73	<0.001	(2.57, 5.33)

In sensitivity analyses, we used population denominators from 2020 instead of 2010 (though 2020 reservation population counts have known undercounting issues - see Discussion). Estimated risk ratios using 2020 denominators were larger, with reservation RRs reaching between 2.08 and 5.84 after adjusting for population density, and borderland RRs reaching between 2.80 and 5.15. See *SI Appendix,* Table S1.

### Sensitivity Analyses.

We first fit a sensitivity analysis adding a fixed effect for Oklahoma, given how much of the state is comprised of reservations. Doing so had a negligible impact; if anything, relative risks for reservation and border lands increased slightly once the Oklahoma fixed effect was added (*SI Appendix,* Table S2).

We also fit models varying the width of the borderlands around reservations. *SI Appendix,* Fig. S1 shows estimated rate ratios for reservations and borderlands as the width of those borderlands changes, from 1 mile to 20 miles. These show that borderland rate ratios were maximized at a width of 11 miles before plateauing: The increased risk associated with being in the borderlands around reservations remained elevated up to at least 20 miles from reservations.

Finally, we evaluated whether our results were solely driven by differential measurement error across geographic areas—i.e., by AIAN deaths being differentially undercounted in areas far from reservations. Results show deaths in areas far from reservations would have to be multiplied by a factor of 2.53 to 4.58 to eliminate the elevated rate ratios in and around reservations (*SI Appendix,* Table S3). That is, between (1–1/2.53=) 60% and (1–1/4.58=) 78% of AIAN police violence deaths far from reservations would have had to have been incorrectly recorded as some other race (and thus be missing from our data) to explain away the elevated rate ratios we observe. Note that we would also have to assume 0% of AIAN deaths were incorrectly identified in and around reservations; otherwise missingness far from reservations would have to be higher still.

That 60 to 78% figure is much higher than rates of racial misattribution of AIAN people estimated by past research, such as 11 to 28% in a nationwide 1996 Indian Health Service report (26); 17% in a Washington state study by AHRQ, of deaths from 1990–2009 (27); or 29% in Oklahoma, for deaths from 1991–2013 (which found misattribution rates were declining over time) (28).

Most recently, a nationwide study compared self-reported vs. death certificate-reported AIAN classification in the Mortality Disparities in American Communities study, using deaths from 2008–2019 (29). For deaths from assault or legal intervention, only 15% were misattributed on death certificates among those who identified only as AIAN (n = 30,500 decedents), or 35% among those who identified as AIAN in combination with another race/ethnicity (n = 58,000)—both much lower than would be required to nullify the inequities we observe.

## Discussion

Fatal police violence against AIAN people is dramatically concentrated in and around reservations, even after adjusting for population density or urbanicity. This does not appear artifactual. Only an implausible level of differential measurement error could explain away observed associations.

These findings will not be a surprise to the Indigenous people living on and near reservations. They echo what communities have tracked, named, and resisted across generations. From the American Indian Movement’s documentation of police killings in the 1960s (30), to the Wounded Knee Occupation (31), to contemporary youth-led protests (32), police violence has been understood as a tool of settler control. Our findings confirm what that history of resistance has already made visible: Fatal police violence is structured, spatial, and concentrated where Indigenous jurisdiction is most constrained.

### Implications for Public Health.

Roughly 1 in every 1800 Native American men will die from fatal police violence if current rates hold (2). We find this mortality risk is multiple times higher in and around reservations, making fatal police violence a major population-level cause of premature death for area residents. Public health agencies lack a coordinated response, both to the deaths themselves and to related community harms that may extend beyond fatalities.

In particular, there is a large body of research demonstrating the long-lasting adverse consequences of loss and trauma under settler colonialism for Indigenous population health (22). This literature and framework align with evidence from research among Black US Americans suggesting that fatal police violence imposes chronic community stress, grief, and hypervigilance (33–35). When this psychological duress occurs during pregnancy, it is sufficiently severe that it can harm perinatal health outcomes, including pregnancy loss, preterm birth, and severe maternal morbidity (36–38). Over time, the cumulative effects of racialized violence and trauma can dysregulate the physiologic stress response, imperiling long-term health and development (39–43). Policing-related stress, heightened by fatal police violence on reservations, may thus widely damage AIAN communities’ well-being across the life course. However, the social, psychological, and physical health impacts of fatal police violence against AIAN peoples remain undermeasured, understudied, and unaddressed. Additional research, prevention, and mitigation strategies are urgently needed.

### Implications for Policy.

To understand potential policy solutions, it is again instructive to follow Indigenous scholars in understanding present-day patterns of fatal police violence against Indigenous peoples as a reflection of settler colonialism. Policing on tribal lands, Rocha Beardall argues, reflects settler systems of social control that perpetuate Indigenous land theft, racialized violence, removal, and political subordination (8, 17). There are clear parallels, for example, between contemporary patterns of policing in and around reservation lands (15, 16), and the historical reservation pass system imposed by the Bureau of Indian Affairs that would arrest Indigenous people if they attempted to leave the reservation without a pass from an Indian agent (8, 44). Today, these systems are institutionalized in the overlapping federal, state, and tribal jurisdictions that structure exposure to law enforcement in and around reservations (13, 45), and in well-documented police harassment at reservation borders (15, 16).

This legacy is also visible in the steady rise of jails and people incarcerated in Indian Country over the past 20 years, despite brief lows during the COVID-19 pandemic (8, 46). As in other jurisdictions, law enforcement is increasingly the primary policy response to public health crises among marginalized peoples, including substance use, interpersonal violence, and mental health conditions, implemented through criminalization and confinement (47). Reliance on carceral institutions as both a component and, at times, a substitute for the social safety net has become a dominant way of governing the lives of marginalized people, including Indigenous people (48, 49).

Policy responses that might lower geographically disproportionate rates of fatal police violence against Indigenous people are needed. Some solutions proposed by non-Indigenous scholars to combat police violence include body cameras, implicit bias trainings, and police force diversification. Unfortunately, evidence to support the potential of these approaches to lower police violence inequities is often negative or disputed (50–55), leaving their ability to resolve the racial and spatial inequities we describe unclear.

Importantly, Indigenous-led alternatives to current carceral practices already exist (56–58). These include 1) restorative and community justice initiatives, including the Aneth Community Court and the Tulalip Tribal Court Healing to Wellness programs; 2) culturally rooted harm-reduction and reintegration supports, including the Muscogee Nation Reintegration Program and school-based prevention such as the GREAT and Attendance Achievement programs; and, 3) to the north in Canada, community-led safety and mutual aid programs, including Bear Clan Patrol neighborhood patrols, Mama Bear Clan overdose response, Ikwe Safe Rides women-led transport, and Drag the Red search and support (56–58). Investments in these Indigenous-led community health solutions and social services, which bolster Indigenous sovereignty, may serve as an alternative policy approach with potential to lower police contact, arrest, and lethal force, in part because some of these interventions seek to obviate law enforcement responses to socioeconomic and mental health crises by providing more care-forward interventions.

### Strengths and Limitations.

Several limitations apply. First, while Mapping Police Violence addresses known undercounting in vital statistics data, it does not capture every incident of fatal police violence (59). Whether uncaptured deaths differ systematically (particularly, by the AIAN identities of decedents or across reservation geographies) is an open question and may cause bias. Second, we lack data on nonfatal police violence, a nascent but critical area where inequities may differ in magnitude (60). Third, we rely on reported race/ethnicity, as captured by MPV. As discussed, this measure differs from true race/ethnicity for a portion of decedents (and in any event would not reflect actual tribal membership). Even so, our sensitivity analyses indicate that bias from racial/ethnic misattribution could not plausibly explain away the inequities we describe here.

Fourth, our small sample of deaths (and even smaller number of block groups with >1 death) preclude us from including state fixed effects because they would cause severe overfitting. Inclusion of fixed effects for specific states, though, did not diminish our observed associations.

Finally, fifth, our data are unable to determine what is driving the geographic inequities we observe. AIAN peoples may be differentially racialized by law enforcement depending on local histories and place-based expectations (61). That is, in practice, officers may read cues including proximity to reservation borders, vehicle plates, language, clothing, and kin or community ties to help determine who they stop and the degree of force they apply (15–17). Geographic inequities may also be driven by the type of officers doing the stopping, given jurisdictional overlap, resulting cross-deputization, and state-specific Public Law 280 agreements (62). Alternatively, geographic inequities in fatal police violence may be primarily driven by structurally imposed poverty, substance use, mental health challenges, and low access to medical care in and around reservations. Our data cannot disaggregate these processes, but underscore the urgent need for future research (and investment in Indigenous health researchers to carry those investigations out).

Our study also has important strengths. We analyze a comprehensive and rigorously fact-checked database of fatal police violence in the United States. Analyses use both single-race and multiracial definitions of AIAN identity, helping to reflect the diversity of AIAN people’s lives. We also explicitly evaluate the impact of modeling and design choices, including how we define reservations’ borderlands and the year from which we draw population denominators, and assess the plausibility of measurement bias.

Counting and categorizing AIAN identity is political and complex, in part because it combines political membership with racialized status. First, federal requirements that each tribal nation articulate and enforce enrollment rules (often based on computed lineal ancestry but sometimes also on tribal connections or knowledge) mean the standards for tribal membership vary (63). Second, pandemic disruptions and rural internet barriers depressed reservation counts and widened already problematic undercounts in the US Census (64)—i.e., geographically specific undercounting varies over time. Third, ancestry versus race reporting keeps many AIAN people off race tallies, including millions who report AIAN ancestry without AIAN race and low dual reporting among multiracial AIAN and White children (64, 65). Given this complexity, the number of people counted as AIAN in the Census is a narrow and likely undercount of the Indigenous population.

### Conclusions.

More broadly, our findings demonstrate the necessity of examining not just whether and at what rates specific groups experience elevated risks of fatal police violence, but also how that violence is geographically distributed and why. These sociologically and geospatially informed analyses can push public health research forward, toward revealing the specific, emplaced policy structures undergirding fatal police violence inequities that—justice demands—must end.

## Materials and Methods

We first created two datasets: a decedent file (where the unit of analysis is AIAN individuals killed by police) to examine decedent characteristics and a US Census block group file to calculate rate ratios via quasi-Poisson regression.

### Outcome: Police Violence Deaths.

Death data were drawn from the Mapping Police Violence database (MPV), spanning from 2013 (the earliest available data) to 2024. We turned to MPV because no comprehensive government database of people killed by law enforcement exists in the United States (4). Current state and federal databases rely on voluntary reporting by police departments and thus miss most cases (5). To fill that gap, MPV serves as an independent data initiative. MPV uses scraping tools to identify and deduplicate potential incidents of fatal police violence from local and national news outlets, then assigns them to human researchers for manual review and data extraction (24). Two researchers independently extract data for each case and resolve disputes. Extracted data include decedents’ demographics, the agencies involved, the cause of death, and its location. Previous research finds approaches like MPVs are superior to federal data systems and capture the vast majority of people killed by police (5).

Importantly, MPV only includes deaths if police directly kill someone while on duty (24); car crashes during a police chase, for example, would be excluded unless police lethally ran into a decedent with their police car. Suicides that occurred while police were present are similarly excluded. This allows us to conservatively study police violence per se (although there are also known racialized, gendered patterns among excluded deaths) (66).

MPV records AIAN deaths separately from deaths of Native Hawaiians and other Pacific Islanders. We also do not hypothesize that AIAN people from the continental United States would be at elevated risk of police violence near Native Hawaiian reservations. We thus exclude the state of Hawai’i from all analyses. In any event, MPV identified 0 AIAN police violence deaths in Hawai’i across the study period.

For modeling, our outcome was cumulative counts of AIAN people killed by police violence, rolled up to US Census block groups.

### Population Counts.

We used block group-level population counts from the 2010 Census to estimate rates of fatal police violence with population denominators. We opted for the 2010 Census to avoid underrepresentation of AIAN people in the 2020 Census, particularly on reservations (67); in sensitivity analyses, however, we use 2020 denominators given their close alignment in time with our study period.

We used two different counts for all analyses: 1) people who selected AIAN only (“single race” analyses) and 2) people who selected AIAN only or in combination with any other racial/ethnic group (“multiracial analyses”). Neither of these represents counts of official tribe members, but using both gives us approximate low and high bounds for the AIAN population.

We note that scholars have debated whether to use population counts as the denominator in analyses of police violence inequities, versus using police stops (or some other proxy for criminality or initial police contact) as the denominator (68). We opt for population denominators because the rate at which people are stopped (a highly discretionary police action strongly shaped by racism) or commit crimes (itself downstream of racialized structural disinvestment) is a mediator in this analysis; controlling for stop rates by using stops as a denominator (even if we had the data to do so) would thus underestimate true inequities and induce mediator-outcome confounding (69, 70), a problem population denominators avoid.

### Exposures and Descriptive Statistics.

**Fig. 1.**
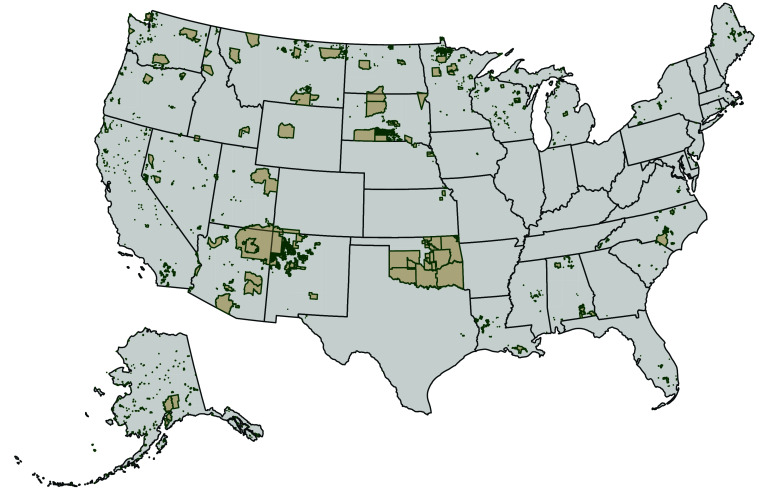
Map of American Indian/Alaska Native (AIAN) reservations in the United States.

Reservation boundaries were drawn from US Census shapefiles (Fig. 1), which we analyzed alongside death data via the statistical programming language R. We first geocoded the location of each death and mapped these against reservation lands. We then created spatial buffers, or border areas, with radii of 5 and 10 miles around each reservation. This enabled us to tabulate the percentage of police violence deaths that occurred within different areas:

(A) reservations
(B) within a 5-mile borderland around reservations
(C) within a >5 to 10-mile borderland around reservations
(D) not near reservations (>10 miles from a reservation).

These areas look something like concentric circles, allowing us to count how many deaths occurred within the center circle (area A), the ring just outside of it (area B), the ring just outside of that (area C), or not within any circle (area D).

We compared these percentages to the percentage of AIAN people living in each of the areas listed above. Populations within each geographic area were approximated by multiplying the population of each block group by the proportion of that block group covered by a reservation or specific area around a reservation.

Finally, we calculated means and SDs for decedent demographics, as well as characteristics of the police officers who killed them. In particular, we examined the reasons police gave for initially stopping decedents, as well as whether they were federal, state, tribal, county, or local/municipal police. Agency coding was performed as per Jahn and Schwartz (62).

### Modeling.

Finally, we fit quasi-Poisson regression models at the block-group level predicting rates of fatal police violence against AIAN people, with population offsets (Eq. **1**). Unadjusted models included only 3 predictors: the proportion of the block group covered by area A (reservation lands), the proportion of the block group covered by area B (the 5-mile borderland around reservations), and the proportion of the block group covered by area C (the borderland between 5 and 10 miles of reservations). In fully adjusted models, we also included, separately, population density (residential population per square mile) or a 9-category USDA classification of rural–urban status at the county level. Exponentiated coefficients represent rate ratios: the multiplicative difference between block groups 100% covered by areas A, B, or C and block groups not near reservations (completely in area D). Block groups with 0 AIAN residents were excluded.

[1] $$\begin{array}{ll} log\left(\frac{deaths}{AIAN\;population}\right) & =\beta_{0}+\beta_{1}\left(Prop.in\;Area\;A\right)\\ & +\beta_{2}\left(Prop.in\;Area\;B\right)+\beta_{3}\left(Prop.in\;Area\;C\right)\\ & +\beta_{4}\left(Pop\;Density\;or\;USDA\;Rurality\right). \end{array}$$

### Sensitivity Analyses.

We completed three sensitivity analyses. First, we added a fixed effect for Oklahoma, since reservations cover huge swaths of the state (Fig. 1). This makes it difficult to disentangle the potential impact of being in/around reservations from the impact of Oklahoma state policies. A fixed effect helps evaluate whether Oklahoma policies skew national results.

Second, our choice of 5 and 10 miles to define reservation borderlands was somewhat arbitrary. We thus fit models in which we varied the width of the borderlands (i.e., within 1 mile of a reservation, within 2 miles, within 3 miles, *et cetera*, through 20 miles) to see how that changed estimated rate ratios. Here, we included as predictors only population density, the proportion of a block group covered by reservations, and the proportion of a block group covered by a borderland buffer of a specific width, yielding 20 models (a 1-mile borderline model, a 2-mile model, etc.).

Finally, we examined whether differential mismeasurement of race/ethnicity could bias results. In particular, AIAN police violence mortality rates could be uniquely underestimated in areas far from reservations: Coroners or journalists may be less likely to correctly identify the race/ethnicity of AIAN decedents the farther from a reservation they die (27, 28, 71–73). This scenario would artificially lower the AIAN mortality rate in comparison block groups (far from reservations), leading to overestimated risk ratios.

To assess this, we calculated crude rate ratios comparing areas in and around reservations (A, B, and C) to areas far from reservations (D). We then multiplied the number of AIAN deaths in Area D by an arbitrary inflation factor and recalculated crude rate ratios, sequentially increasing the inflation factor from 1 by increments of 0.01 until augmented crude rate ratios were null (equaled 1). This allowed us to evaluate how extreme the differential mismeasurement would have to be (the percentage of deaths far from reservations that would have to have been missed) to make null any positive associations we observe.

## Supplementary Material

Appendix 01 (PDF)

## Data Availability

Previously published data were used for this work. [Death data is publicly available from Mapping Police Violence: https://mappingpoliceviolence.org (74). Denominator data and reservation and tract shapefiles are freely, publicly available from the US Census].
