# Supplementary material for: Heightened risk of fatal police violence in and around reservations for American Indian/Alaska Native peoples in the United States
Source: Proc Natl Acad Sci U S A. 2026 Mar 9;123(11):e2521002123. doi: 10.1073/pnas.2521002123 (PMC12994187; doi:10.1073/pnas.2521002123)
Supplement: Supplementary file 1 — Appendix 01 (PDF) [file pnas.2521002123.sapp.pdf]

SUPPLEMENTAL FILES

for

Heightened risk of fatal police violence in and around reservations for American Indian/Alaska Native peoples in the United States

Schwartz, Rocha Beardall, Jahn

CONTENTS

Table S1. Estimated fatal police violence rate ratios from quasi-Poisson regression models using 2020 Census population denominators

Figure S1. Sensitivity analysis showing how estimated police violence mortality rate ratios change depending on how reservation borderlands are defined (i.e., depending on their width), by AIAN population denominator

Table S2. Estimated fatal police violence rate ratios from quasi-Poisson regression models adjusting for population density and a fixed effect for the state of Oklahoma

Table S3. Inflation factors needed to bring crude rate ratios (based on Table 2) to 1, by multiplicatively inflating the number of observed deaths in areas far from reservations

Table S1. **Estimated fatal police violence rate ratios from quasi-Poisson regression models using 2020 Census population denominators**

Denominator	Place	Unadjusted			Adjusted for Pop. Density			Adjusted for USDA Rurality		
		RR	*p*	*95% CI*	*RR*	*p*	*95% CI*	*RR*	*p*	*95% CI*
Single-Race AIAN	Reservation	2.86	<0.001	(2.16, 3.80)	2.08	<0.001	(1.53, 2.83)	2.09	<0.001	(1.47, 3.00)
	Buffer: 5 mi	3.31	<0.001	(2.35, 4.62)	2.79	<0.001	(1.97, 3.92)	2.80	<0.001	(1.90, 4.09)
	Buffer: 10 mi	3.47	<0.001	(2.35, 5.03)	3.19	<0.001	(2.15, 4.66)	3.36	<0.001	(2.18, 5.09)
Multiracial AIAN (Single or Multiracial)	Reservation	6.94	<0.001	(5.38, 8.95)	5.84	<0.001	(4.46, 7.65)	5.05	<0.001	(3.72, 6.87)
	Buffer: 5 mi	5.47	<0.001	(4.03, 7.38)	5.15	<0.001	(3.78, 6.97)	4.83	<0.001	(3.46, 6.69)
	Buffer: 10 mi	4.65	<0.001	(3.28, 6.50)	4.57	<0.001	(3.21, 6.41)	4.66	<0.001	(3.19, 6.70)

Figure S1. **Sensitivity analysis showing how estimated police violence mortality rate ratios change depending on how reservation borderlands are defined (i.e., depending on their width), by AIAN population denominator**

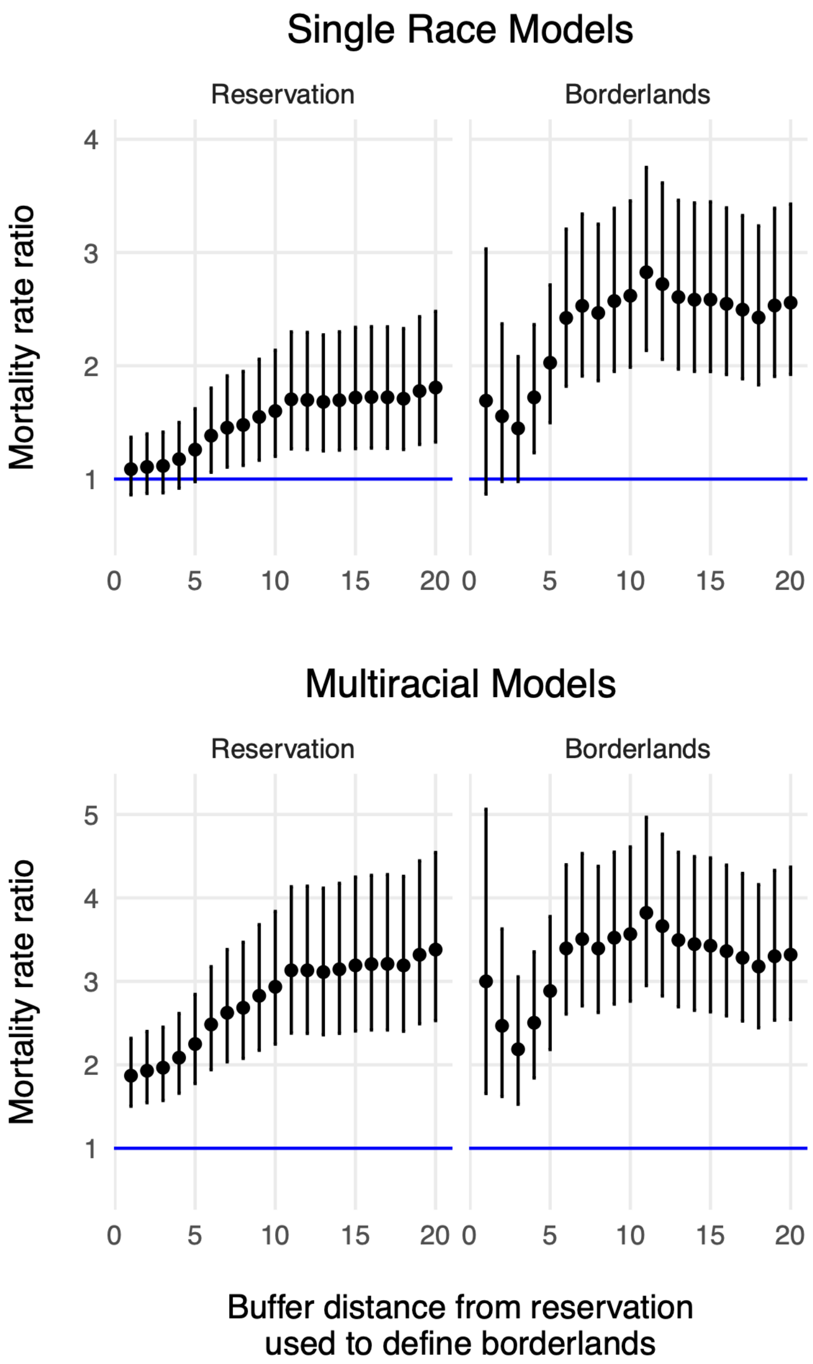

Table S2. **Estimated fatal police violence rate ratios from quasi-Poisson regression models adjusting for population density and a fixed effect for the state of Oklahoma**

Denominator	Place	Adjusted for Population Density & an OK Fixed Effect		
		RR	*p*	*95% CI*
Single-Race AIAN	Reservation	1.65	0.001	(1.21, 2.26)
	Buffer: 5 mi	2.44	<0.001	(1.75, 3.38)
	Buffer: 10 mi	2.93	<0.001	(2.01, 4.19)
Multiracial AIAN (Single or Multiracial)	Reservation	3.36	<0.001	(2.52, 4.49)
	Buffer: 5 mi	3.58	<0.001	(2.63, 4.83)
	Buffer: 10 mi	3.70	<0.001	(2.62, 5.16)

Table S3. Inflation factors needed to bring crude rate ratios (based on Table 2) to 1, by multiplicatively inflating the number of observed deaths in areas far from reservations

Place	Inflation factor needed to reduce rate ratio to 1, by place and population denominator	
	Single-Race	*Multiracial*
On a reservation	2.53	4.58
Within 5 mi	2.46	3.54
Within 10 mi	3.22	3.99

Note: Inflation factors were assessed by first calculating crude rate ratios from Table 2, comparing reservations or areas around reservations (Areas A, B, and C) to areas far from reservations (Area D). We then multiplied the number of AIAN deaths by an inflation factor only for Area D and recalculated crude rate ratios, increasing the inflation factor from 1 by increments of 0.01 until augmented crude rate ratios were null (equaled 1). We present here the inflation factors that brought augmented crude rate ratios closest to 1.00.
